# Rice-eating pattern and the risk of metabolic syndrome especially waist circumference in Korean Genome and Epidemiology Study (KoGES)

**DOI:** 10.1186/1471-2458-13-61

**Published:** 2013-01-22

**Authors:** Younjhin Ahn, Seon-Joo Park, Hye-kyoung Kwack, Mi Kyung Kim, Kwang-Pil Ko, Sung Soo Kim

**Affiliations:** 1 Division of Epidemiology and Health Index, Center for Genome Science, National Institute of Health, Center for Disease Control and Prevention, Chungcheongbuk-do, South Korea; 2 Department of Preventive Medicine, College of Medicine, Hanyang University, Seoul, South Korea; 3 Department of Preventive Medicine, Graduate School of Medicine, Gachon University, Incheon, South Korea

**Keywords:** Rice, Beans, Multi-grains, Metabolic syndrome (MetS), Postmenopausal women

## Abstract

**Background:**

Metabolic syndrome poses a serious health threat in Asian countries. Rice is a staple food in Korea, and carbohydrate intake is associated with the risk of MetS. We hypothesized that various rice-eating patterns in a carbohydrate-based diet would have different effects on the risk of MetS.

**Methods:**

Participants were 26,006 subjects enrolled in the Korean Genome and Epidemiology Study between 2004 and 2006. They were classified into four dietary patterns - white rice, rice with beans, rice with multi-grains, and mixed based on their food frequency questionnaire responses. We compared metabolic risk traits according to the rice-eating patterns.

**Results:**

Nutrients consumption and the presence of MetS risk factors differed according to rice-eating patterns. In men odds ratio(OR) for central obesity was slightly elevated in mixed group(1.18). In women, the risk for central obesity and abnormal fasting glucose were lower in the rice with beans group (adjusted OR =0.79, 0.83 respectively) and central obesity in rice with multi-grains(adjusted OR=0.91) than the white rice group. In postmenopausal women, ORs for central obesity (0.78) and abnormal fasting glucose (0.75) in the rice with beans group and ORs for central obesity (0.83), abnormal HDL-cholesterol (0.87) and MetS(0.85) in the rice with multi-grains group was lower than those in white rice group. In premenopausal women, the risk for central obesity (OR=0.77) was reduced in the rice with beans group.

**Conclusion:**

The risk for MetS was lower in the rice with beans and rice with multi-grains groups compared with the white rice group, particularly in postmenopausal women.

## Background

Metabolic syndrome (MetS) is characterized by a combination of disturbed glucose and insulin metabolism, central obesity, dyslipidemia, and hypertension [1]. MetS is a risk factor for type 2 diabetes and cardiovascular disease [1-4] and presents a serious health threat that is on the rise in Western and Asian countries [5-7]. The prevalence of MetS has been reported to be 34% among adults aged 20 years and over in the United States [8], and 32.5% for men and 31.8% for women over 30 years of age in Korea [9]. The cause of MetS is unclear but it is thought to be associated with genetic, environmental, and dietary factors [10-14].

The amount of carbohydrate intake affects blood glucose and insulin. Furthermore, the quality of the carbohydrate determined by the composition of diet is associated with metabolic consequences. Several studies have reported associations between dietary carbohydrate quality/quantity and lipid profile [15], risk of diabetes [16], and insulin action [11].

Koreans consume 306.58 g of carbohydrate, which contributes 63.4% of their total daily energy consumption in 2005 [9]. The main source of carbohydrate is rice. Koreans consume rice two or three times a day as a staple food and it supplies 37.9% of their total energy consumption [9]. White rice is usually cooked alone or together with beans and multi-grains in Korea.

In the present study, to determine whether various rice-eating patterns in a carbohydrate-based diet would have different effects on the risk of MetS, we compared the metabolic risk traits according to rice-eating patterns classified using the food frequency questionnaire (FFQ) data collected in the Korean Genome and Epidemiology Study (KoGES), a large population-based cohort study of Koreans.

## Methods

### Subjects

The Korean Genome and Epidemiology Study (KoGES) was launched in 2001 with a population based cohort of 2 cities each in rural and urban area of central part of Korea. The main objectives of this study were to establish national genomic cohort and to examine causal relationship between the genetic and environmental factors associated with major diseases such as diabetes mellitus, hypertension, obesity, and MetS in Korea. The size and scope of the project were gradually expanded and it encompasses 7 cohort studies mainly targeting 40 to 69 years age group. KoGES has recruited more than 240,000 participants by the end of 2011. The written informed consent was obtained from each participant.

We analyzed data of participants who joined KoGES between November 2004 and December 2006 in this study. Of those, 40,254 subjects had available dietary data of food frequency questionnaire (FFQ). The records with extremely low or high energy intake (<800 or >4,000 kcal for men and <500 or >3,500 kcal for women) were excluded (*n* = 978).

Subjects with previous diagnosed hypertension, hyperlipidemia, diabetes mellitus, or cancer (any type) were excluded from the study (*n*=11,842) because of the possibility of dietary habit change after diagnosis. Subjects with no waist circumference, triglyceride, high-density lipoprotein (HDL) cholesterol, blood pressure (BP), or fasting glucose data (*n*=1,428) were further excluded from our study. Finally, 26,006 subjects were included for the analysis of this study.

### Data collection

Participants were recruited in 28 health examination centers across the country (Chonbuk National University Hospital, Chonnam National University Hospital, Chunchoen Sacred Heart Hospital, Daegu Catholic University Medical Center, Dankook University Hospital, Dobong-Gu Health Center, Dong-A University Medical Center, Ehwa Womans University Mokdong Hospital, Gangdong Health Center, Gimje Public Health Center, Green Hospital, Gyeongju Health Center, Hallym University Sacred Heart Hospital, Inha University Hospital, Inje University Sanggye Paik Hospital, Jechoen Public Health Center, Jungnang Health Center, Jungwon-Gu Health Center, Kangbuk Samsung Hospital, Konkuk University Chungju Hospital, Korea University Ansan Hospital,Kosin University Gospel Hospital, Kyungpook National University Hospital, Seongbuk Health Center, Sujeong-Gu Health Center, Ulsan University Hospital, Yeoncheon-Gun Health Center and County Hospital, Yonsei University Hospital Occupational Health Center). They originally visited for their health check-up and voluntarily agreed to participate in the KoGES. Questionnaires including age, gender, education level (<=6 years, 7–12 years, 12 years <), smoking status (never/ex-/current), drinking status (non/ex-/current), dietary supplement use (yes/no), regular exercise (yes/no), and menopause status (yes/no) and FFQ were administered by trained interviewers.

Blood pressures (BPs) and anthropometric measurement were obtained by trained personnel. Waist circumference was measured midway between the inferior margin of last rib and crest of the ilium in a horizontal plane in 0.1cm unit. Blood pressures were measured by a trained technician using a mercury sphygmomanometer. Subjects had 10 minutes resting before taking the BP measurements. Two readings were taken on the subject’s dominant arm while in the sitting position with a 5-minute interval between readings. The BP values reported here are the mean of the two measurements.

Participants were asked to fast more than 8 hours before examination and fast venous blood samples were drawn at study site. Fasting glucose, triglycerides, and HDL cholesterol were assessed using a 7600–210 automatic analyzer (Hitachi, Tokyo, Japan).

### Dietary data

Dietary intake was assessed using the 103-item FFQ (Additional file 1: Appendix 1). The development and validation of the questionnaire has been described in previous reports [17,18]. Briefly, the FFQ was designed using dietary data obtained from the Korea Health and Nutrition Examination Survey (KHANES) 24-h recall data in 1998. Food items were selected using a data-based approach, and several seasonal foods were added to the final food list.

The frequency of servings was classified into nine categories: never or seldom, once a month, two or three times a month, one or two times a week, three or four times a week, five or six times a week, once a day, twice a day, or three times or more a day. The portion size was categorized as small, medium, or large. Participants were requested to answer the length of time they used seasonal food items as 3, 6, 9, or 12 months. The FFQ has been validated in 124 subjects using a 12-day diet record.

The nutrient intake from each food item in the FFQ was determined by the weight derived from the consumption frequency and portion size of each food item. The daily nutrient intake of each individual was calculated as the sum of the nutrient intake from each food item. The nutrient and isoflavone compositions used in the calculation were based on the Food Composition Table [19,20].

Glycemic load was calculated by multiplying carbohydrate content of each food item, which was based on the reported consumption frequency and portion size by its glycemic index. The glycemic load value for one day was calculated as the sum of all food items. The overall dietary glycemic index was calculated by dividing the average daily glycemic load by the average daily carbohydrate intake. The glycemic index value for each food item was obtained from the International Tables of Glycemic Index [21], the online Glycemic Index Database maintained by the University of Sydney [22], and a previous report that listed the glycemic index of Korean foods [23]. The reference of glycemic index values was glucose (glycemic index for glucose = 100).

### Classification of subjects

Total of 26,006 subjects were included for analysis and classified into four groups according to their response on the rice-eating pattern. Before asking consumption frequency and amount of cooked rice, we asked ‘What kind of cooked rice do you usually eat? (white rice only/rice with other foods/mix two types)’ and then ‘What kind of foods do you mainly eat cooked rice with? (beans/multi-grains)’ Rice-eating patterns were defined as ‘eating white rice group’ (*n*=6,136), ‘eating rice with beans’ (*n*=2,589), ‘eating rice with multi-grains’ (*n*=12,440), and ‘eating rice in mixed’ (*n*=4,841).

MetS was defined by the presence of three or more of the following five components according the Adult Treatment Panel using waist circumference for Asians [24,25]: central obesity (≥ 90 cm for men or ≥ 80 cm for women); reduced HDL cholesterol (<40 mg/dL for men or <50 mg/dL for women); hypertriglyceridemia as defined by an elevated triglyceride level(≥ 150 mg/dL); elevated BP as defined by systolic blood pressure (SBP) ≥ 130 mmHg or diastolic blood pressure (DBP) ≥ 85 mmHg; and raised fasting glucose (≥100 mg/dL).

Smokers were defined as having smoked at least 400 cigarettes during their lifetime and divided into current- or ex-smokers based on their current smoking status. Drinkers were classified as drinkers or ex-drinkers in terms of current alcohol intake, and nondrinker was defined as being unable or unwilling to drink during their lifetime. Subjects who reported using any dietary supplements were classified as supplement users. Subjects were classified as regular exercisers or not if they answered to do exercise until sweaty regularly.

### Statistical analysis

General/metabolic characteristics and nutrients intake are expressed as the means ± standard deviations (SD) for continuous variables and as frequencies or percentages for categorical variables. The effect of the rice-eating patterns was assessed using analysis of variance (ANOVA) for continuous variables and chi-square test for categorical variables. Multiple comparisons of the mean were performed using Scheffe’s test. Triglyceride, HDL cholesterol, BP, and fasting serum glucose were log-transformed to improve normality.

The relationships between rice-eating patterns and MetS were examined using logistic regression. Odds ratios (ORs) and 95% confidence intervals (CIs) were calculated. Adjustments were made for age, total energy intake, education level, supplement use, exercise, drinking, and smoking. We analyzed the effects of rice-eating patterns by gender because the prevalence of MetS and reported risk factors differ by gender.

All statistical analyses were performed using SAS v9.13 (SAS Institute Inc., Cary, NC, USA) and a *P*-value of 0.05 was considered to be statistically significant.

The design of study was reviewed and approved by the Institutional Review Board of Korea Centers for Disease Control and Prevention.

## Results

Characteristics of the participants according to their rice-eating patterns are shown in Table 1. The mean age of men was highest in the rice with beans group (54.9 years old). The 35 to 44 year-old participants ate more white rice alone and 45 to 54 year-old participants were more distributed in mixed group. Over 55 year-old men were more abundant in rice with beans group. Higher proportions of current smokers, drinkers, people who did not take supplements and did not exercise were observed in white rice group. The habits of participants in the rice with beans and rice with multi-grains groups were healthier than those of participants in the white rice group. Similar to the finding for men, the mean age of the women in the rice with beans group was older than those of the other groups (51.8 years). Younger (< 55 years) participants were in mixed group and older participants were in rice with beans group. The proportion of participants who exercised was higher in the rice with multi-grains groups and the proportion of dietary supplements eaters was higher in mixed group than in the white rice-alone group. The proportion of postmenopausal women was higher in rice with beans group.

**Table 1:** Participants’ characteristics according to rice-eating patterns

	**Total (*n *= 26,006)**	**White rice group (*n *= 6,136)**	**Rice with other foods group**		**Mixed group (*n *= 4,841)**	***P***
			**Rice with beans (*n *= 2,589)**	**Rice with multi-grains (*n *= 12,440)**		
*Men*						
No. of subjects	7,561	2,386	699	2,991	1,485	
Age (years)						
mean ± SD	52.2 ± 8.8	50.6 ± 8.7 ^c^	54.9 ± 9.1^a^	53.3 ± 8.8^b^	51.1 ± 8.0^c^	<0.0225
Age group, years, *n* (%)						
35–44	1,728 (22.9)	711 (29.8)	97 (13.9)	561 (18.8)	359 (24.2)	<0.0001
45–54	2,997 (39.6)	940 (39.4)	253 (36.2)	1,150 (38.4)	654 (44.0)	
55–64	2,029 (26.8)	545 (22.8)	216 (30.9)	889 (29.7)	379 (25.5)	
64–75	807 (10.7)	190 (8.0)	133 (19.0)	391 (13.1)	93 (6.3)	
Education levels, years, *n* (%)						
<= 6	2,186 (29.5)	708 (30.2)	245 (35.8)	905 (30.8)	328 (22.7)	<0.0001
7~12	2,843 (38.4)	900 (38.5)	260 (37.9)	1,143 (38.8)	540 (37.4)	
12 <	576 (32.1)	733 (31.3)	180 (26.3)	894 (30.4)	576 (39.9)	
Smoking status, *n* (%)						
Nonsmoker	2,250 (29.9)	674 (28.3)	224 (32.2)	921 (30.9)	431 (29.2)	<0.0001
Ex-smoker	2,829 (37.5)	825 (34.7)	276 (39.7)	1,151 (38.6)	577 (39.0)	
Current smoker	2,457 (32.6)	880 (37.0)	195 (28.1)	912 (30.5)	470 (31.8)	
Drinking status, *n* (%)						
Nondrinker	1,522 (20.2)	443 (18.6)	163 (23.4)	639 (21.4)	277 (18.7)	0.0108
Ex-drinker	552 (7.3)	161 (6.8)	48 (6.9)	235 (7.9)	108 (7.3)	
Current drinker	5,456 (72.5)	1,771 (74.6)	486 (69.7)	2,105 (70.7)	1,094 (74.0)	
Exercise, *n* (%)						
No	3,648 (48.4)	1,334 (56.5)	319 (45.7)	1,305 (43.7)	680 (45.9)	<0.0001
Yes	3,895 (51.6)	1,035 (43.5)	379 (54.3)	1.679 (56.3)	802 (54.1)	
Supplement use, *n* (%)						
No	5,407 (71.7)	1,847 (77.8)	478 (68.5)	2,059 (68.9)	1,023 (69.1)	<0.0001
Yes	2,132 (28.3)	526 (22.2)	220 (31.5)	929 (31.1)	457 (30.9)	
Waist circumference (cm)	85.4 ± 7.3	85.1 ± 7.4 ^b^	84.9 ± 7.5 ^b^	85.6 ± 7.3 ^ab^	86.0 ± 7.3 ^a^	0.0004
Triglyceride (mg/dL)	149.6 ± 108.8	150.4 ± 105.6	155.3 ± 121.1	148.4 ± 108.3	148.2 ± 108.8	
HDL cholesterol (mg/dL)	52.8 ± 12.3	52.8 ± 12.3	52.7 ± 12.0	52.9 ± 12.2	52.9 ± 12.4	
SBP (mmHg)	124.8 ± 15.3	124.3 ± 15.1 ^b^	126.3 ± 16.2 ^a^	125.1 ± 15.4 ^ab^	124.4 ± 14.9 ^ab^	0.0122
DBP (mmHg)	78.6 ± 10.0	78.4 ± 9.7	79.2 ± 10.7	78.6 ± 10.0	78.5 ± 9.9	
Fasting glucose (mg/dL)	95.0 ± 18.9	94.5 ± 18.3	95.4 ± 17.6	95.4 ± 18.5	95.0 ± 21.2	
No. of subjects with MetS (%)	1,486 (19.4)	454 (19.0)	136 (19.5)	594 (19.9)	284(19.1)	
*Women*						
No. of subjects	18,445	3,750	1,890	9,449	3,356	
Age (years)						
mean ± SD	50.4 ± 7.7	49.6 ± 7.7^c^	51.8 ± 7.7^a^	50.7 ± 7.7^b^	49.6 ± 7.3^c^	<0.0001
Age group, years, *n* (%)						
35–44	4,621 (25.1)	1,089 (29.0)	364 (19.2)	2,248 (23.8)	920 (27.4)	<0.0001
45–54	8,840 (47.9)	1,759 (46.9)	909 (48.1)	4,487 (47.5)	1,685 (50.2)	
55–64	3,917 (21.2)	693 (18.5)	487 (25.8)	2,121 (22.4)	616 (18.4)	
64–75	1,067 (5.8)	209 (5.6)	130 (6.9)	593 (6.3)	135 (4.0)	
Education levels, years, *n* (%)						
<= 6	7,549 (41.9)	1,624 (44.4)	867 (47.2)	3,824 (41.4)	1,234 (37.6)	<0.0001
7~12	7,356 (40.8)	1,426 (39.0)	716 (39.0)	3,825 (41.4)	1,389 (42.3)	
12 <	658 (17.3)	607 (16.6)	254 (13.8)	1,558 (17.2)	658 (20.1)	
Smoking status, *n* (%)						
Nonsmoker	17,716 (96.7)	3,536 (95.2)	1,830 (97.3)	9,111 (96.9)	3,239 (97.1)	<0.0001
Ex-smoker	243 (1.3)	65 (1.7)	29 (1.5)	112 (1.2)	37 (1.1)	
Current smoker	371 (2.0)	114 (3.1)	22 (1.2)	175 (1.9)	60 (1.8)	
Drinking status, *n* (%)						
Nondrinker	9,428 (63.3)	2,288 (61.6)	1,271 (67.5)	5,923 (63.1)	2,023 (60.9)	<0.0001
Ex-drinker	379 (2.5)	105 (2.8)	50 (2.7)	224 (2.4)	67 (2.9)	
Current drinker	5,129 (34.2)	1,321 (35.6)	562 (29.8)	3,246 (34.5)	1,207 (36.2)	
Exercise, *n* (%)						
No	8,982 (48.9)	2,213 (59.4)	882 (46.8)	4,270 (45.4)	1,617 (48.3)	<0.0001
Yes	9,383 (51.1)	1,515 (40.6)	1,001 (53.2)	5.139 (54.6)	1,728 (51.7)	
Supplement use, *n* (%)						
No	10,439 (56.8)	2,371 (63.7)	1,061 (56.4)	5,142 (54.6)	1,865 (55.8)	<0.0001
Yes	7,926 (43.2)	1,355 (36.3)	822 (43.6)	4,274 (45.4)	1,475 (44.2)	
Menopause, *n* (%)						<0.0001
Post-	8,856 (48.0)	1,636 (43.6)	1,065 (56.4)	4,665 (49.4)	1,490 (44.4)	
Pre-	9,589 (52.0)	2,114 (56.4)	825 (43.6)	4,784 (50.6)	1,866 (55.6)	
Waist circumference (cm)	78.0 ± 7.8	78.3 ± 7.8	77.7 ± 7.5	78.0 ± 7.8	77.8 ± 7.9	
Triglyceride (mg/dL)	107.7 ± 70.7	109.0 ± 72.6	108.9 ± 69.1	107.9 ± 71.0	105.2 ± 68.3	
HDL cholesterol (mg/dL)	58.6 ± 12.7	58.4 ± 12.8	58.2 ± 12.5	58.8 ± 12.8	58.7 ± 12.5	
SBP (mmHg)	118.7 ± 15.7	118.8 ± 16.0 ^b^	120.2 ± 15.6 ^a^	118.7 ± 15.7 ^b^	117.6 ± 5.4 ^c^	<0.0001
DBP (mmHg)	74.0 ± 10.0	74.1 ± 10.1 ^ab^	74.8 ± 9.9 ^a^	74.1 ± 10.0 ^b^	73.2 ± 9.8 ^c^	<0.0001
Fasting glucose (mg/dL)	90.6 ± 16.2	90.8 ± 14.3	89.8 ± 13.5	90.7 ± 15.9	90.1 ± 20.0	
No. of subjects with MetS (%)	2,999 (16.3)	625 (16.7)	325 (17.2)	1,572 (16.6)	477 (14.2)	

For differences among rice-eating patterns using ANOVA with Scheffé’s test for data that are means ± SDs(continuous variables) and chi-square test for data that are percentages(categorical variables).

MetS component variables were compared according to rice-eating patterns. Means of WC and SBP in men and those of SBP and DBP in women were significantly different among groups.

Table 2 shows nutrients consumption among the rice-eating pattern groups. The dietary glycemic index was highest in the white rice group. The rice with beans group showed the highest consumption of protein, fiber and total isoflavones intake. Total energy and energy from cooked rice, percentage of energy from cooked rice, carbohydrate intake, and dietary glycemic load were the highest in rice with multi-grains group. Dietary characteristics by rice eating patterns were similar in both genders.

**Table 2:** **Comparisons of nutrients intake according to gender and rice-eating patterns**^***1***^

**Nutrient/food group intake**	**Total (*n *= 26,006)**			**White rice group (*n *= 6,136)**			**Rice with other foods group**						**Mixed group (n=4,841)**		
							**Rice with beans (*n *= 2,589)**			**Rice with multi-grains (*n *= 12,440)**					
*Men*															
Total energy (kcal)	1,920	±	541.6	1,845	±	536.3^c^	1,849	±	504.8^bc^	2,001	±	547.6^a^	1,911	±	532.3 ^b^
Energy from cooked rice (kcal)	1,073	±	313.7	982	±	284.1^c^	1,033	±	288.9 ^b^	1,161	±	320.6^a^	1,059	±	303.7 ^b^
Energy % from cooked rice (%)	57.6	±	14.8	55.1	±	15.0^c^	57.4	±	14.5^b^	59.6	±	14.4^a^	57.0	±	14.3 ^b^
Protein (g)	63.5	±	24.0	60.7	±	24.0^b^	65.7	±	21.9^a^	65.2	±	24.1^a^	63.3	±	24.5 ^a^
Fat (g)	30.6	±	17.0	30.9	±	17.3	31.9	±	16.1	30.0	±	16.8	30.8	±	17.2
Carbohydrate (g)	341.0	±	92.1	324.2	±	88.9^c^	319.3	±	85.0^c^	360.0	±	93.8^a^	338.1	±	89.4 ^b^
Fiber (g)	6.0	±	2.9	5.3	±	2.8^d^	7.0	±	2.8^a^	6.4	±	2.9^b^	5.8	±	2.8 ^c^
Glycemic Index	56.9	±	3.1	57.2	±	3.2^a^	56.1	±	3.1^d^	56.9	±	3.1^c^	57.0	±	3.1 ^b^
Glycemic Load	193.6	±	51.3	184.8	±	48.9^b^	178.7	±	45.9^c^	204.6	±	52.4^a^	192.7	±	50.4 ^ab^
Total isoflavones (mg)	11.5	±	9.3	8.6	±	7.3^c^	25.5	±	9.7^a^	11.0	±	8.3^b^	11.0	±	7.9 ^b^
*Women*															
Total energy (kcal)	1,742	±	522.0	1,637	±	509.4^c^	1,694	±	515.0^b^	1,796	±	524.2^a^	1733	±	511.8 ^b^
Energy from cooked rice (kcal)	944.0	±	320.5	847.6	±	281.3^c^	909.9	±	299.9^b^	998.0	±	332.5^a^	919.0	±	307.0 ^b^
Energy % from cooked rice (%)	55.6	±	16.2	53.9	±	16.9^c^	55.3	±	16.0^b^	56.8	±	16.1^a^	54.4	±	15.8 ^bc^
Protein (g)	58.4	±	23.1	54.0	±	22.9^c^	61.1	±	22.8^a^	59.7	±	23.1^a^	58.1	±	22.9 ^b^
Fat (g)	26.3	±	15.2	26.0	±	16.2^b^	27.4	±	14.1^a^	26.1	±	15.1^b^	26.7	±	14.9 ^ab^
Carbohydrate (g)	313.1	±	91.9	292.0	±	86.7^c^	297.3	±	89.9^c^	325.7	±	93.0^a^	310.5	±	89.7 ^b^
Fiber (g)	6.2	±	3.2	5.4	±	3.0^d^	7.1	±	3.2^a^	6.4	±	3.2^b^	6.0	±	3.1 ^c^
Glycemic Index	56.3	±	3.6	56.7	±	3.8^a^	55.5	±	3.6^c^	56.2	±	3.5^b^	56.3	±	3.5 ^b^
Glycemic Load	176.0	±	51.0	165.2	±	47.8 ^c^	164.5	±	48.3^c^	183.0	±	51.8^a^	174.9	±	49.7 ^b^
Total isoflavones (mg)	11.8	±	10.5	8.4	±	8.6^c^	23.9	±	11.1^a^	11.0	±	9.7^b^	10.8	±	9.2 ^b^

1. Mean ± SD.

2. Different alphabet shows that means are significantly different among rice-eating patterns using ANOVA with Scheffé’s test for multiple comparisons (P<0.05).

Table 3 shows odds ratios of MetS and component risk factors. In men, the risk for central obesity was maintained after adjustment for several confounding factors (adjusted OR in model 3=1.18) in mixed group. The risk for high BP was greater in rice with beans (OR = 1.27) and rice with multi-grains (OR = 1.14) groups compared with the white rice group, however, the associations were not significant after adjusting for confounding variables. No other risk factors or MetS were associated with rice-eating patterns. In women, the odds ratios for central obesity and high fasting glucose were significantly lower after adjustment in rice with beans group(adjusted OR in model 3=0.79, 0.83 respectively). The risk for high BP was elevated in the rice with beans and the significance was disappeared after adjustment. The ORs for central obesity(adjusted OR in model 3=0.91) and hypertriglycemia (adjusted OR in model 2=0.90) in the rice with multi-grains group with reference to the white rice group were significantly lower after adjustment. The risk for BP was lowered in mixed group and the significance was remained after adjustment (adjusted OR in model=0.86). The OR for MetS in the rice with beans and the rice with multi-grains groups was significantly lower in model 2 (adjusted OR= 0.89 and 0.82, respectively). The OR for MetS of mixed group showed significantly lower in all models.

**Table 3:** Odds ratios (95% CIs) for metabolic syndrome and the individual components according to rice-eating patterns and gender

**Metabolic syndrome traits**	**White rice group**	**Rice with other foods group**				**Mixed group**	
		**Rice with beans**		**Rice with multi-grains**			
	**Ref**	**OR**	**95% CI**	**OR**	**95% CI**	**OR**	**95% CI**
Men							
Waist circumference							
Model 1	1	0.97	0.80-1.18	1.07	0.95-1.21	1.26	1.05-1.39
Model 2	1	0.93	0.77-1.13	1.00	0.88-1.13	1.18	1.02-1.36
Model 3	1	0.91	0.75-1.11	0.98	0.87-1.12	1.18	1.02-1.36
Triglyceride							
Model 1	1	0.92	0.77-1.10	0.93	0.83-1.04	0.98	0.86-1.12
Model 2	1	1.00	0.83-1.19	0.96	0.86-1.08	0.98	0.86-1.24
Model 3	1	1.06	0.88-1.27	1.01	0.90-1.13	1.02	0.89-1.17
HDL cholesterol							
Model 1	1	1.15	0.91-1.46	0.95	0.82-1.12	1.00	0.83-1.21
Model 2	1	1.12	0.89-1.43	0.94	0.80-1.10	1.00	0.83-1.21
Model 3	1	1.13	0.89-1.45	0.95	0.81-1.12	1.02	0.84-1.24
Blood pressure							
Model 1	1	1.27	1.07-1.51	1.14	1.02-1.27	1.04	0.91-1.19
Model 2	1	1.15	0.96-1.36	1.06	0.94-1.18	1.03	0.90-1.17
Model 3	1	1.16	0.98-1.38	1.09	0.97-1.22	1.08	0.94-1.24
Fasting glucose							
Model 1	1	1.03	0.86-1.24	1.07	0.95-1.20	0.99	0.85-1.14
Model 2	1	0.98	0.82-1.19	1.03	0.92-1.17	0.98	0.85-1.13
Model 3	1	0.96	0.80-1.16	1.02	0.91-1.16	0.99	0.86-1.15
Metabolic syndrome							
Model 1	1	1.03	0.83-1.27	1.06	0.92-1.20	1.01	0.85-1.19
Model 2	1	1.01	0.81-1.25	1.02	0.87-1.17	0.99	0.84-1.17
Model 3	1	1.01	0.81-1.26	1.03	0.89-1.19	1.03	0.87-1.22
Women							
Waist circumference							
Model 1	1	0.94	0.84-1.05	1.00	0.92-1.08	0.94	0.86-1.04
Model 2	1	0.78	0.69-0.87	0.88	0.81-0.95	0.92	0.83-1.01
Model 3	1	0.79	0.70-0.90	0.91	0.84-0.99	0.97	0.88-1.08
Triglyceride							
Model 1	1	1.10	0.96-1.27	0.98	0.89-1.08	0.91	0.81-1.03
Model 2	1	0.97	0.84-1.12	0.90	0.82-0.99	0.90	0.80-1.03
Model 3	1	0.98	0.85-1.13	0.92	0.83-1.02	0.93	0.82-1.05
HDL cholesterol							
Model 1	1	1.02	0.90-1.15	0.95	0.88-1.04	0.94	0.85-1.04
Model 2	1	0.96	0.85-1.09	0.92	0.85-1.01	0.94	0.85-1.04
Model 3	1	0.98	0.86-1.11	0.96	0.88-1.09	0.98	0.88-1.09
Blood pressure							
Model 1	1	1.15	1.02-1.30	1.02	0.93-1.11	0.84	0.75-0.93
Model 2	1	0.98	0.86-1.12	0.93	0.85-1.02	0.83	0.74-0.93
Model 3	1	1.01	0.89-1.15	0.96	0.88-1.05	0.86	0.77-0.97
Fasting glucose							
Model 1	1	0.91	0.79-1.05	0.97	0.88-1.07	0.90	0.80-1.02
Model 2	1	0.84	0.72-0.97	0.92	0.83-1.02	0.90	0.80-1.02
Model 3	1	0.83	0.71-0.96	0.94	0.85-1.04	0.91	0.80-1.04
Metabolic syndrome							
Model 1	1	1.04	0.90-1.20	1.00	0.90-1.10	0.83	0.73-0.94
Model 2	1	0.87	0.74-1.00	0.89	0.80-0.99	0.82	0.72-0.94
Model 3	1	0.89	0.76-1.04	0.93	0.84-1.04	0.87	0.75-0.99

Model 1 : Not adjusted, Model 2 : adjusted for age and total energy intake, Model 3 : adjusted for age, total energy, education level, supplement use, exercise, drinking, and smoking.

Metabolic syndrome was defined as the presence of ≥3 of 5 components.

Table 4 shows the multivariate adjusted OR for MetS and its components in women by menopause states. Because menopause is associated with increased risk of MetS, and waist circumference is also increased after menopause, we analyze the MetS risks by rice-eating pattern before and after menopause separately. The ORs for central obesity and abnormal glucose control were significantly reduced in rice with beans group, the OR for central obesity in rice with multi-grains group, and the ORs for central obesity, HDL abnormality and MetS in mixed group were lower in postmenopausal women. However, in premenopausal women, the OR for central obesity was reduced only in the rice with beans group.

**Table 4:** Multivariate adjusted odds ratios (95% CIs) for metabolic syndrome and individual components of metabolic syndrome according to rice-eating patterns in women

**Metabolic syndrome traits**	**White rice group**	**Rice with other foods group**				**Mixed group**	
		**Rice with beans**		**Rice with multi-grains**			
	**Ref**	**OR**	**95% CI**	**OR**	**95% CI**	**OR**	**95% CI**
Postmenopausal women							
Waist circumference	1	0.78	0.66-0.92	0.83	0.73-0.94	0.88	0.75-1.03
Triglyceride	1	0.92	0.76-1.12	0.88	0.77-1.02	0.86	0.72-1.03
HDL cholesterol	1	0.90	0.76-1.08	0.87	0.76-0.99	0.82	0.70-0.97
Blood pressure	1	1.08	0.91-1.29	1.00	0.88-1.14	0.87	0.74-1.03
Fasting glucose	1	0.75	0.61-0.92	0.92	0.80-1.07	0.89	0.74-1.07
Metabolic syndrome	1	0.86	0.71-1.04	0.85	0.73-0.98	0.74	0.62-0.89
							
Premenopausal women							
Waist circumference	1	0.77	0.64-0.93	0.97	0.87-1.10	1.02	0.89-1.17
Triglyceride	1	1.02	0.80-1.31	0.95	0.81-1.12	0.93	0.77-1.13
HDL cholesterol	1	1.03	0.85-1.24	1.05	0.93-1.02	1.11	0.96-1.29
Blood pressure	1	0.90	0.73-1.11	0.92	0.80-1.05	0.86	0.73-1.01
Fasting glucose	1	0.93	0.74-1.18	0.95	0.82-1.10	0.97	0.81-1.17
Metabolic syndrome	1	0.88	0.66-1.17	1.06	0.89-1.27	1.03	0.83-1.28

Odds ratios were adjusted for age, total energy, education level, supplement use, exercise, drinking, and smoking.

The subjects with hormone replacement therapy were excluded among postmenopausal women.

Metabolic syndrome was defined as the presence of ≥3 of 5 components.

## Discussion

The present study compared the effect of rice-eating patterns on MetS. We categorized the subjects into four rice-eating groups: white rice alone, rice with beans, rice with multi-grains, and mixed group. We analyzed the effects of rice-eating patterns by gender because the prevalence of MetS and reported risk factors differ by gender [5,26]. We found that the risk for metabolic disorders was lower in the rice with beans and rice with multi-grains groups compared with the white rice group, particularly in postmenopausal women. The characteristics such as lifestyle habits, nutrient consumption, and metabolic indices differed according to rice-eating patterns.

The one of main characteristics of Korean diet is high proportion of carbohydrate intake because rice is the staple food. Korean eats rice only or with other foods such as beans or other grains. White rice can be characterized as a refined grain because it is polished during processing and the bran and germ are entirely removed. The rice with beans group showed some features of bean consumption, and the rice with multi-grains group showed the benefits of whole grain. The third Korea National Health and Nutrition Examination Survey (KNHANES III) reported grains such as barely, sorghum, glutinous rice, Job’s tears, oats, millet and glutinous millet [9].

We found reduced risks for central obesity, dyslipidemia, and MetS in participants in the rice with multi-grains group compared with those in the white rice group. The rice with beans diet showed a beneficial effect on waist circumference and fasting glucose control. However, we could not find the BP lowering effect of rice with bean diet.

The most distinctive result was about central obesity. Means of WC of men in rice with multi-grains group and mixed group were higher than that of white rice group and odds ratio for central obesity was also elevated. However, means of WC in women were not significantly different among rice-eating groups, ORs for WC in rice with beans group and rice with multi-grains group were reduced. Especially in rice with beans group, both pre and post menopause women had low risk for central obesity.

We believe that the reduced ORs for MetS and the MetS components observed in rice with beans and multi-grains groups were the effect of soy protein and/or isoflavones in the beans group and cereal fibers and various components of unpolished grains in the rice with multi-grains group. The presence of dietary fiber, vitamins and minerals, phenolic acid, and phytoestrogen may contribute to the protective effect of whole grains [27]. Whole grain intake is associated with a reduction in the risk for metabolic disease [28-31].

Soybean provides protein, fiber, and isoflavones. Clinical trials have shown positive effects of soy and soyfoods on total cholesterol, low-density lipoprotein (LDL) cholesterol, HDL cholesterol, and improvement in glycemic control [32-34]. Several studies have shown that soy isoflavones reduce the risk of coronary heart disease or cardiovascular disease by controlling blood lipid levels [33,35,36].

The study had some unavoidable weaknesses. Because we use baseline data, this study had a limitation of a cross-sectional study. This kind of study cannot provide information of changes over time and explain the causal relationship between risk factor and outcome. However, as cohort study is going on, disease outcomes would be ascertained. It provides the chance to investigate the risk of not only long term effect of mass carbohydrate consumption but also dietary pattern related to socio-psychological tendency for health and diseases.

Food frequency questionnaire had a limitation of ingredient assuming [30]. We supposed that ‘rice with multi grains’ have whole grain effect but amount and kind of grains must be assumed in the ‘rice with multi grains’ item of food frequency questionnaire. The amount of beans was also estimated. These might have the association between diet and disease weakened.

The strength of this study was that metabolic risk factors were compared by rice-eating pattern in large scale data. And we could show the positive effects on health by rice eating with beans and multi-grains even in high carbohydrate consuming population.

We applied strict inclusion criteria in the present study to clearly identify the effect of rice-eating patterns on MetS. To avoid misclassification, the subjects themselves indicated their rice-eating patterns. It could clarify the feature of each pattern group. Furthermore, because disease may alter eating habits or lifestyle, we also excluded subjects with a diagnosis of a major disease. This reduces the available data but can make the effect of exposure in cross-sectional study relatively clearer.

The effect of rice-eating pattern on the metabolic components of MetS differed according to gender. We found a benefit of eating rice with beans or multi-grains in women; however, little difference in metabolic variables was observed among the rice-eating patterns in men. Because trends of nutrient intake by rice-eating pattern were similar in men and women (Table 2) and life-style factors were statistically controlled, gender difference of metabolic risks is due to intrinsic factor like hormone.

This finding is consistent with that of Kim et al. [26] who reported differences between men and women in their study on dietary carbohydrate and MetS, and with suggestion of Sun et al. [37] that genetic and/or hormonal influences may cause sexual dimorphism. Moreover, the effect of rice-eating patterns differed between pre- and postmenopausal women in the Kim et al. study [26]. The primary metabolic symptom in postmenopausal women is central obesity, which is likely associated with changes in the androgen-to-estrogen ratio after menopause [38]. Our results indicated that the beneficial effects of beans and multi-grains were the greatest in postmenopausal women, who lack protective hormones.

The results of this study suggested a desirable rice eating style. Rice is a main food in Asian countries and rice consumption is increasing in US [39]. Eating rice with beans or other grains can provide the variety on rice consumption and apply to increase whole grains consumption rather than refined grains as a partly replacement method in western countries.

## Conclusions

The present study investigated the effects of rice-eating pattern on MetS indices in a large-scale data set. We conclude that eating rice with beans or multi-grains was associated with healthy effects and a reduction in metabolic risk factors, particularly in waist circumference and in postmenopausal women, although our findings reflect a relatively short-term effect on dietary habit. This study is a cross-sectional study. These results cannot explain the causal relationship of health effect by rice-eating patterns, prospective studies are needed.

## Abbreviations

BP: Blood Pressure; FFQ: Food Frequency Questionnaire; HDL cholesterol: High-Density Lipoprotein Cholesterol; KoGES: Korean Genome and Epidemiology Study; MetS: Metabolic Syndrome; OR: Odds Ratios; SBP: Systolic Blood Pressure; DBP: Diastolic Blood Pressure.

## Competing interests

The authors declare that they have no competing interests.

## Author’s contributions

The author responsibilities were as follows - YA designed, initiated, developed analytic strategy, interpreted the results, and drafted the manuscript; SP and HK contributed to collection and analyzing the data, interpretation of results, and drafting of the manuscript. MK and KK contributed to preparation of data and interpretation of results; SSK designed and interpreted the results and was a principal investigator of this study. All authors read and reviewed the final manuscript.

## Pre-publication history

The pre-publication history for this paper can be accessed here:

http://www.biomedcentral.com/1471-2458/13/61/prepub

## Supplementary Material

Additional file 1: Appendix 1 Food list of food frequency questionnaire used in Korean Genome and Epidemiology Study. Click here for file
