# Supplementary material for: Rice-eating pattern and the risk of metabolic syndrome especially waist circumference in Korean Genome and Epidemiology Study (KoGES)
Source: BMC Public Health. 2013 Jan 22;13:61. doi: 10.1186/1471-2458-13-61 (PMC3680034; doi:10.1186/1471-2458-13-61)
Supplement: Additional file 1: Appendix 1 — Food list of food frequency questionnaire used in Korean Genome and Epidemiology Study. [file 1471-2458-13-61-S1.docx]

Additional file 1. Food list of food frequency questionnaire used in Korean Genome and Epidemiology Study

| **Food or food group** | **Food list** |
| --- | --- |
| Rice and rice cake | Cooked rice (White rice), Cooked rice(Rice with other food), Parched cereal powder, Rice cake (plain rod shape)/rice cake with soup, Other rice cakes |
| Noodles | Ramyon, noodles with soup, Chajangmyon/Jambbong, buckwheat vermicelli/buckwheat noodle, Dumpling/Dumpling with soup |
| Bread | Loaf bread/Sandwich/Toast, Bread with small red beans, Other breads, Pizza/Hamburger, Cereals, corn flakes |
| Sugar and fat | Cakes/Chocopie, Cookie/Cracker/Sanck, Candy/Chocolate, Jam/Honey/Butter/Margarine, coffee sugar, coffee cream |
| Potatoes | Potatoes, sweet potatoes, starch vermicelli, starch jelly |
| Legumes and Nuts | Nuts, legumes, Soup and Stew with soybean paste/soybean paste, tofu, soybean milk, |
| Kimchi | Korean cabbage kimchi, Kakduki/small radish kimchi, kimchi, Radish with water, Other Kimchi |
| Vegetables | Radish/Salted radish, Korean cabbages/Korean cabbage soup, Spinach, Lettuce, Perilla leaf, Vegetables wrap/Vegetable salad, Other green vegetables, Deoduck/Doraji(kinds of white root), Bean sprouts, Bracken/Sweet potato stalk/Stem of taro, Pepper leaves/Chamnamul/Asterscaber, Crown daisy/Leek/Water dropwort, Cucumber, Carrot/Carrot juice, Onion, Green pepper, Pumpkin, immature, Pumpkin,mature/Pumpkin juice, Korean style pickles |
| Mushrooms | Oyster mushroom, other mushrooms |
| Fruits | Strawberry, Muskmelon/Melon, Watermelon, Peach/Plum, Banana, Persimmon, hard/Persimmon, dried, Tangerine, Pear/Pear juice, Apple/Apple juice, Orange/Orange juice, Grape/Grape juice, Tomato/Cherry tomato/Tomato juice |
| Meat | Pork belly, Roasted pork, Braised pork, Ham/Sausage, Edible viscera, Steak/Roasted beef, Dog meat, Fried chicken/Chicken stew, Beef soup, Beef soup with vegetables |
| Eggs | Eggs |
| Fish and Seafood | Sushi, Mackerel/Pacific saury/Spanish mackerel, Hair tail, Eel, Yellow croaker/Sea bream/Flat fish, Alaska Pollack, Cuttlefish/Octopus, Dried anchovy, Tuna, canned, Salt-fermented fish, Clam/Whelk, Oyster, Crab, Shrimp, Fish paste/Crab flavored |
| Seaweeds | Laver, kelp/sea mustard |
| Milk | Milk |
| Dairy products | Yoghurt, ice cream, cheese |
| Beverages | Coffee, Green tea, carbonated drinks, Other drinks |
